# HDAC5-mediated PRAME regulates the proliferation, migration, invasion, and EMT of laryngeal squamous cell carcinoma via the PI3K/AKT/mTOR signaling pathway

**DOI:** 10.1515/med-2023-0665

**Published:** 2023-03-09

**Authors:** Lei Yu, Huan Cao, Jian-Wang Yang, Wen-Xia Meng, Chuan Yang, Jian-Tao Wang, Miao-Miao Yu, Bao-Shan Wang

**Affiliations:** Department of Otorhinolaryngology, The Second Hospital of Hebei Medical University, Shijiazhuang, Hebei, China

**Keywords:** laryngeal squamous cell carcinoma, PRAME, HDAC5, PI3K/AKT/mTOR signaling pathway, biomarker, prognosis

## Abstract

Laryngeal squamous cell carcinoma (LSCC) is an aggressive and lethal malignant neoplasm with extremely poor prognoses. Accumulating evidence has indicated that preferentially expressed antigen in melanoma (PRAME) is correlated with several kinds of cancers. However, there is little direct evidence to substantiate the biological function of PRAME in LSCC. The purpose of the current study is to explore the oncogenic role of PRAME in LSCC. PRAME expression was analyzed in 57 pairs of LSCC tumor tissue samples through quantitative real-time PCR, and the correlation between PRAME and clinicopathological features was analyzed. The result indicated that PRAME was overexpressed in the LSCC patients and correlated with the TNM staging and lymphatic metastasis. The biological functions and molecular mechanism of PRAME in LSCC progression were investigated through *in vitro* and *in vivo* assays. Functional studies confirmed that PRAME facilitated the proliferation, invasion, migration, and epithelial–mesenchymal transition of LSCC cells, and PRAME also promoted tumor growth *in vivo*. HDAC5 was identified as an upstream regulator that can affect the expression of PRAME. Moreover, PRAME played the role at least partially by activating PI3K/AKT/mTOR pathways. The above findings elucidate that PRAME may be a valuable oncogene target, contributing to the diagnosis and therapy of LSCC.

## 1 Introduction

Laryngeal squamous cell carcinoma (LSCC) has been found as one of the most common malignant neoplasms among head and neck cancer [1]. Moreover, the annual morbidity and mortality of LSCC tend to increase worldwide due to the complex and multifaceted characteristics [2,3]. For instance, early symptoms of LSCC are so atypical that about 60% of LSCC patients are diagnosed at an advanced or metastatic stage [4]. Currently, despite a variety of interventions, such as early prevention, chemotherapy, radiotherapy, and surgical resection, the 5-year survival rate of LSCC patients still remains relatively low [5]. Thus, the molecular regulatory mechanisms of the development of LSCC and the promising targets for precise therapy of LSCC should be further identified.

In recent years, our group has been devoted to exploring the pathogenesis of LSCC from clinical study to molecular basis. Previously, we reported several potential biomarkers such as TGF-beta, HO-1, and CEACAM1 that were closely related to the head and neck squamous cell carcinoma [6,7,8]. Deeper investigation in our group revealed that several potential long non-coding RNAs such as LINC00668, lncRNA SSTR5-AS1, and MIR155HG that also make great contribution to the pathogenesis of LSCC [8,9,10]. These data suggested that the pathogenesis of LSCC is a complex, involving numerous molecular targets. Preferentially expressed antigen in melanoma (PRAME) is a human melanoma antigen originally identified by cDNA expression cloning targeting melanoma-reactive cytotoxic T cells [11]. Gene expression profiling has revealed that the upregulation of PRAME in melanoma can be distinguished from benign melanocytic proliferation in clinical studies [12,13]. Moreover, PRAME is also involved in other malignancies, such as non-small-cell lung cancer [14], esophageal cancer [15], ovarian carcinoma [16], and breast carcinoma [17]. However, whether PRAME is involved in the development of LSCC remains unclear.

Epigenetic regulation, like ubiquitin, methylation, and acetylation, plays an important biological role in many tumors, including laryngeal carcinoma [18]. Several studies suggested that PRAME is associated with the epigenetic regulation of acetylation in human malignancies like seminomas and melanoma [19,20]. More importantly, histone acetylation under the regulation of the conserved enzymes of histone acetyltransferases (HATs) and histone deacetylases (HDACs) has been confirmed as one of the most typical epigenetic modifications in the regulation of gene expression [21]. There is growing evidence that abnormal expression of HDAC5 plays a role in cancer cell proliferation, metastasis, and invasion, thus contributing to the development and progression of various human tumors (e.g., lung, colon, breast) [22]. Furthermore, there is evidence that HDAC5 frequently shows low expression levels in human malignant tumors [23,24]. However, no specific role for HDAC5 in LSCC correlated with PRAME has been demonstrated thus far. Thus, we hypothesized that PRAME may be involved in regulating the expression of HDAC5, contributing to the pathogenesis of LSCC.

PI3K/Akt pathway is one of the most important signaling pathways that is often reported to be involved in human cancers [25]. Recently, Ghafouri-Fard et al. reported that PI3K/AKT pathway has the central role in head and neck cancers like squamous cell carcinomas through the regulation of malignant behavior such as cell proliferation, cell survival, and gene or protein expression [26]. However, whether the PI3K/Akt pathway is regulated by the PRAME in the development of LSCC is still unclear. Therefore, the aim of the present study was to identify the expression of PRAME in LSCC patients associated with poor prognosis and survival rate. Furthermore, exploring the potential roles of PRAME and its upstream of HDAC5 and exploring its downstream regulatory of PI3K/Akt pathway in the pathogenesis of LSCC [6,8,10] are also the main goals of the present study. Here, we propose and identify the hypothesis “HDAC5-mediated PRAME regulates the malignant behavior of LSCC via the PI3K/AKT/mTOR signaling pathway” in this study.

This study demonstrated that PRAME is overexpressed in LSCC tissues. High expression of PRAME is also significantly correlated with poor prognosis and survival rate in the LSCC patients. *In vitro*, we have found that PRAME overexpression has the ability to stimulate LSCC-related AMC-HN-8 and TU177 cell proliferation, migration, invasion, and epithelial–mesenchymal transition (EMT), and knock-down of PRAME can reduce the proliferative, migratory and invasive capabilities of TU177. *In vitro*, we have also found that HDAC5 as a potential upstream regulator might mediate PRAME expression, contributing to the progression of LSCC. *In vitro* and *in vivo*, our data further revealed that the proliferation, invasion, and migration of LSCC cells that are induced by PRAME may be at least partially dependent on the activation of PI3K/AKT/mTOR signaling pathways. The above findings elucidate the function of PRAME in LSCC development and progression and provide the new evidence for PRAME as an oncogene that probably serves as a valuable target for the diagnosis and management of LSCC.

## 2 Materials and methods

### 2.1 Patients and specimens

A total of 57 pairs of LSCC tumor tissues and corresponding normal tissues were provided by the Second Hospital of Hebei Medical University (Hebei, China) from October 2017 to September 2019. In this study, none of the LSCC patients received chemotherapy, radiotherapy, or other anti-tumor treatment before surgery. All of the tissue specimens were frozen and stored at −80°C at the Institute of Otolaryngology of Hebei Medical University to perform further experiments. All clinical features were acquired from the hospital records and pathological diagnoses, as listed in Tables 1 and 2.

**Table 1:** Basic clinical information statistics of 57 patients of LSCC

Clinicopathological characteristics	PRAME high expression	PRAME low expression
Age	58.22 ± 8.32	60.33 ± 6.24
Male	29(100%)	28(100%)
Alcohol	17(58.62%)	19(67.86%)
Smoking	26(89.65%)	24(82.76%)
TNM		
T		
T1	2(6.90%)	10(35.71%)
T2	7(24.14%)	7(25.00%)
T3	10(34.48%)	8(28.57%)
T4	10(34.48%)	3(10.71%)
N		
N0	15(51.72%)	23(82.14%)
N1	6(20.70%)	3(10.71%)
N2	8(27.59%)	2(7.14%)
M		
M0	29(100%)	28(100%)
M1	0	0
Stage		
Ⅰ	2(6.90%)	10(35.71%)
Ⅱ	4(13.79%)	4(14.29%)
Ⅲ	7(24.14%)	10(35.71%)
Ⅳ	16(55.17%)	4(14.29%)
Differentiation		
High	16(55.17%)	21(75.00%)
Middle	9(31.03%)	5(17.86%)
Low	4(13.79%)	2(7.14%)

**Table 2:** Correlation of PRAME expression with clinicopathological characteristics of LSCC

Clinicopathological characteristics	PRAME high expression	PRAME low expression	*P* value
Age <65	7	3	0.297
Age ≥65	22	25	
Alcohol			
Yes	17	19	0.586
No	12	9	
Smoking			
Yes	26	24	0.706
No	3	4	
T			
Ⅰ + Ⅱ	9	17	0.034*
Ⅲ + Ⅳ	20	11	
N			
0	15	23	0.024*
1+2	14	5	
Stage			
Ⅰ + Ⅱ	8	14	0.106
Ⅲ + Ⅳ	21	14	
Differentiation			
High	16	21	0.167
Middle+Low	13	7	

*P < 0.05.


**Ethics approval and consent to participate:** All procedures in this study were performed were in agreement with the ethical standards of the Research Committee of Hebei Medical University (Hebei, China) as well as with the Declaration of Helsinki (2008). The approval of this study was obtained from the Ethics Committee of Hebei Medical University and the Second Hospital of Hebei Medical University. Informed consent was obtained from all patients.

### 2.2 Cell culture and reagents

The human LSCC cell lines of AMC-HN-8 and TU177 were purchased from Beijing Beina Chuanglian Institute of Biotechnology (Beijing, China). The AMC-HN-8 cells were cultured in Dulbecco’s modified Eagle’s medium (DMEM), containing 10% foetal bovine serum (FBS). The TU177 cells were cultured in RPMI-1640 medium, supplemented with 10% FBS. All of the above cell culture reagents were provided by GIBCO (Thermo Fisher Scientific, Inc). Accordingly, the culture of two cells was performed in a humidified incubator with 5% CO_2_ atmosphere at 37°C (Thermo Fisher Scientific, Inc). The PI3K inhibitor (LY294002) and mTOR inhibitor (Rapamycin) were purchased from MCE (New Jersey, USA). LSCC cells were incubated with LY294002 or Rapamycin to perform functional assays and Western blot assay.

### 2.3 RNA extraction and quantitative real-time PCR assay

Total RNA was extracted from LSCC specimens and cell lines of AMC-HN-8 and TU177 with Eastep^®^Super Total RNA Extraction Kit (Promega, USA) following the experimental instructions. RNA reverse transcription into cDNA was performed with Transcriptor First Strand cDNA Synthesis Kit (Roche, Germany). A GoTaq^®^qPCR Master Mix (Promega, USA) was used for the quantitative real-time PCR (qRT-PCR). Relative expression was normalized using the 2^−∆∆Ct^ method and the glyceraldehyde-3-phosphate dehydrogenase (GAPDH) was assigned into the endogenous control. Primer sequences were as follows:

PRAME, forward: 5′-CAGGACTTCTGGACTGTATGGT-3′;

reverse: 5′-CTACGAGCACCTCTACTGGAA-3′.

GAPDH, forward: 5′-CTCACCGGATGCACCAATGTT-3′;

reverse: 5′-CGCGTTTCACAATGTTCAT-3′.

All the samples were run in triplicate.

### 2.4 Gene transfection

AMC-HN-8 and TU177 were grown in six-well plates and transfected with 3 μg overexpression of PRAME (sinobiological, China) or 3 μg the PcDNA3.1 vector (Invitrogen, USA) using 6 μl Lipofectamine^®^2000 (Invitrogen, USA) in accordance with the manufacturer’s instructions when the cells had 75–80% confluence. Moreover, the empty vector, as control, was transfected under the same condition. In addition, small interfering RNA (siRNA) was adopted to knock-down PRAME expression in TU177. The siRNA of PRAME (siRNA sequence: 5′-GGTCATGCTGACCGATGTA-3′) and negative control (si-NC) were obtained by RiboBio (Guangzhou, China). After the transfection for 48 h, the transfection efficiency was determined through qRT-PCR, and the cells were harvested and employed in subsequent experiments. The expression of PRAME in the treated cells was examined using qRT-PCR as well as western blot assay.

### 2.5 Cell proliferation assay

The proliferation ability of cells was examined through 3-(4,5-dimethylthiazol-2-yl)-5-(3-carboxymethoxyphenyl)-2-(4-sulfophenyl)-2H-tetrazolium inner salt (MTS) assay. AMC-HN-8 and TU177 transfected at 2 × 10^3^ cells/well were cultured in 96-well plates containing 100 μl of complete medium, respectively. 20 µl MTS reagent was introduced into the respective well, followed by the incubation of the plates for 2.5 h at 37°C according to the instructions of the CellTiter96^®^AQueousOne Solution Cell Proliferation Assay Kit (Promega, USA). Furthermore, the optical density (OD) was examined at 490 nm using a Spark^®^ multimode microplate reader (Mod: SPARK 10 M, TECAN, Switzerland). Proliferation rates were examined at 0, 24, 48, 72, and 96 h after the transfection. Each assay was performed in triplicate.

### 2.6 Colony formation assay

A total of 2 × 10^3^ AMC-HN-8- and TU177-transfected cells were seeded into the respective well of a six-well plate and incubated at 37°C in a humidified incubator containing 5% CO_2_. Ten days later, the cell colonies were rinsed gently with PBS in triplicate, fixed with 4% paraformaldehyde for 20 min, and then stained with 0.5% crystal violet for 20 min. Lastly, the number of colonies formed with more than 50 cells per well was calculated using a microscope (CKX53, Olympus Corp.) at 200× magnification.

### 2.7 Wound healing assay

In total, 5 × 10^5^ AMC-HN-8 and TU177 transfected cells were seeded into the respective well of a six-well plate and were cultured at 37°C overnight in a humidified incubator containing 5% CO_2_. When the cell monolayers had 100% confluence, a straight wound line was made by a scratch using a 200 μl pipette tip. Subsequently, the images at the same position of the respective well were captured. The relative distance between cell migration and the scratched area was measured at 0, 24, and 48 h, respectively, under a fluorescence microscope (CKX53; Olympus Corp). The experiment was repeated in triplicate.

### 2.8 Cell migration and invasion assays

Cell migration and invasion abilities were examined through transwell assay (Corning Costar, USA). For the migration assay, a total of 1 × 10^5^ cells in 100 μl culture medium free of FBS were seeded into the upper chamber, followed by supplementation with 650 ul of culture medium with 10% FBS, in the bottom chamber. Following the incubation for 24 h at 37°C and the removal of the transwell chamber, the samples were rinsed with PBS in triplicate, fixed with 4% paraformaldehyde for 20 min and subsequently stained with 0.5% crystal violet for 20 min. For the invasion assay, the membrane was coated with 50 µl Matrigel to develop a matrix barrier first. Subsequently, the same protocol was employed for transwell assay. Lastly, the number of cells that experienced migration or invasion was observed and calculated in five microscopic fields selected randomly by a microscope (CKX53, Olympus Corp.) at a fixed 200× magnification.

### 2.9 Western blot assay

By lysing the cells AMC-HN-8 and TU177 transfected with the overexpression of PRAME or the PcDNA3.1 empty vector, RIPA lysis buffer (Solarbio, China) was adopted for protein extraction, supplemented with PMSF (Solarbio, China) as well as with a protease inhibitor cocktail (Promega, USA). The above step was followed by ultrasonication (Thermo FB120, USA) for higher acquisition efficiency. The protein concentration was examined using the BCA Protein Assay Kit (Generay, China). Protein lysates were then mixed with SDS-PAGE loading buffer (Solarbio, China) and boiled at 95°C for 5 min. Equal amounts of protein (20 µg) were employed for electrophoresis on 10% sodium dodecyl sulfate–polyacrylamide gel electrophoresis and probed with the following primary antibodies. The signals were visualized with enhanced chemiluminescence (vazyme, China) using ChemiDoc™ XRS + (Bio-Rad, USA). All primary antibodies used are listed in Table 3.

**Table 3:** The information for primary antibodies used in western blot analysis

Primary antibody	Dilution concentration	Supplier	Code
PRAME	1:1,000	Abcam	ab219650
N-cadherin	1:5,000	Proteintech	Cat No. 22018-1-AP
E-cadherin	1:5,000	Proteintech	Cat No. 20874-1-AP
Vimentin	1:5,000	Proteintech	Cat No. 10366-1-AP
SNAI1	1:2,000	Proteintech	Cat No. 26183-1-AP
ZEB1	1:2,000	Proteintech	Cat No. 21544-1-AP
β-Catenin	1:10,000	Proteintech	Cat No. 17565-1-AP
p-AKT	1:1,000	CST	4060
p-mTOR	1:1,000	CST	5536
GAPDH	1:10,000	Proteintech	Cat No. 60004-1-Ig
β-Actin	1:6,000	abcam	ab227387
Goat Anti-RabbitIg G(H + L)	1:10,000	Proteintech	Cat No. SA00001-2

### 2.10 Bioinformatic analysis

The GEO online database (https://www.ncbi.nlm.nih.gov/gds/?term=) (e.g., GSE143224, GSE117005, GSE84957, GSE59652, GSE59102, and GSE51985) was adopted to obtain the gene matrix expression profiles of LSCC (Table 4 for specific information). Genes from the above database with log2 (fold change) (log2FC) > 1 and adjusted *P* < 0.05 were considered DEGs. All genes in each dataset were sorted by logFC and integrated using the RobustRankAggreg R package. The GEPIA online database (http://gepia.cancer-pku.cn/) was adopted to analyze the PRAME expression in an enlarged sample size. In addition, a Kaplan–Meier plot (http://kmplot.com/) was employed to analyze the correlation of the expression of PRAME with the overall survival (OS) rate of LSCC patients. The NCBI online database (https://www.ncbi.nlm.nih.gov/) was applied to analyze the expression levels of PRAME in various normal tissues. To further explore the potential mechanism underlying the function of PRAME, Kyoto Encyclopedia of Genes and Genomes (KEGG) pathway analyses were conducted for the prediction of the downstream signaling pathway.

**Table 4:** Details of the Gene Expression Omnibus (GEO) LSCC data sets

Reference	Tissue	GEO	Platform	Normal	Tumor	Biotype
Nicolau et al. (2020) [27]	LSCC	GSE143224	GPL5175	11	14	mRNA
Liu et al. (2020) [28]	LSCC	GSE117005	GPL20115	5	4	mRNA
Feng et al. (2016) [29]	LSCC	GSE84957	GPL17843	9	9	mRNA
Shen et al. (2014) [30]	LSCC	GSE59652	GPL13825	7	6	mRNA
Wilson (2014) [31]	LSCC	GSE59102	GPL6480	13	25	mRNA
Lian et al. (2013) [32]	LSCC	GSE51985	GPL10558	3	10	mRNA

### 2.11 Animal xenograft model

An animal xenograft model was employed to examine the effect of PRAME on tumor growth *in vivo*. A total of 20 male nude (BALB/c nude Crlj) mice (age: 6 weeks old; weight: 18−22 g) were purchased from Beijing Vital River Laboratory Animal Technology Co., Ltd. (Animal Permit NO: SCXK [Jing] 2016-0011). The nude mice were randomly assigned into four groups, including the empty vector group; the transiently transfected PRAME group; the stably transfected PRAME group; and the LY294002 (an inhibitor of PI3K) group (*n* = 5 in the respective group). 1 × 10^7^ AMC-HN-8 cells transfected with overexpressed PRAME were mixed with 100 μl culture medium free of FBS, followed by the subcutaneous injection into the right flanks of the BALB/c nude mice. The tumor volume was documented every 3 days with a caliper by the formula: (*A* × *B* ^2^)/2, where *A* denotes the maximum diameter and *B* represents the diameter perpendicular to *A*. Four weeks later, all nude mice were sacrificed, and the tumor xenografts were harvested immediately and weighed. The animal study was conducted at the Institute of Otolaryngology of Hebei Medical University following the NIH guidelines. All procedures of animal experiments were approved by the Hebei Medical University and were conducted in agreement with international guidelines for the maintenance and care of laboratory animals.

### 2.12 Statistical analysis

Statistical analysis was conducted using SPSS21.0 software (SPSS, Chicago, IL, USA). The qRT-PCR results are expressed as the mean ± standard deviation (S.D), and significance analysis between different groups was conducted using Student’s *t*-test and chi-square test. All statistical tests were two-sided, and *P* < 0.05 indicated a difference achieving statistical significance. GraphPad Prism7.0 (GraphPad Software Inc., La Jolla California USA) was used for relevant diagrams.

## 3 Results

### 3.1 The expression of PRAME and its clinical significance in the LSCC patients

**Figure 1:**
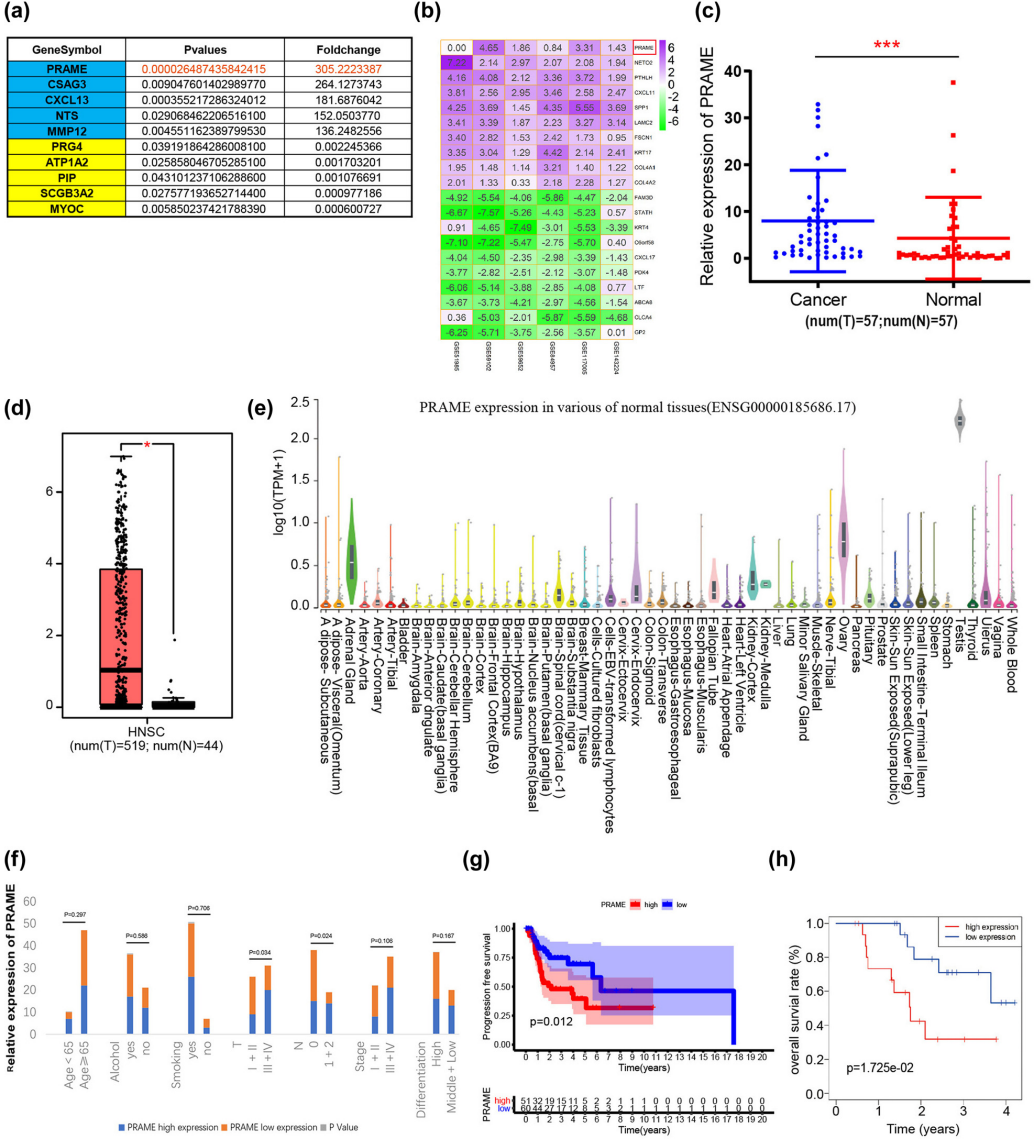
The expression of PRAME and its clinical significance in the LSCC patients. (a) PRAME, as the experimental mRNA, was selected through the microarray analysis for the further experiments. (b) The expression level of PRAME in transcriptome-related microarray datasets of LSCC. (c) PRAME expression was significantly upregulated compared with corresponding normal tissues. (d) PRAME mRNA levels were significantly higher in HNSC compared with paired non-tumor tissues, cited from GEPIA. (e) The expression levels of PRAME (ENSG00000185686.17) in various normal tissues, cited from NCBI. (f) A high level of expression of PRAME was correlated with the TNM staging (*P* < 0.05) as well as with lymphatic metastasis (*P <* 0.05). (g and h) Kaplan–Meier analysis showed that the progression free survival rate (*P =* 0.012) and the overall survival rate (*P =* 1.725 × 10^−02^) of patients in the PRAME high expression group were shorter than those of the PRAME low expression group (*P < 0.05, **P < 0.01, ***P < 0.001).

Microarray analysis was performed to investigate the mRNA expression levels in comparison between four pairs of LSCC tissues and corresponding normal tissues from our sample collected in hospital (Figure 1a). Results were shown as fold-change and *P* values versus normal control, and PRAME was identified as the greatest fold change of 305.222 in upregulated DEGs. Moreover, we used the GEO database to further validate the expression of PRAME in the LSCC. The GEO online database (https://www.ncbi.nlm.nih.gov/gds/?term=) including GSE143224, GSE117005, GSE84957, GSE59652, GSE59102, and GSE51985 were applied to obtain the gene matrix expression profiles of LSCC using the RobustRankAggreg R package (Figure 1b). As we anticipated, the identification of PRAME expression in LSCC tissues showed that its expression was significantly upregulated compared with corresponding normal tissues (Figure 1c). In addition, a similar expression pattern of PRAME in an expanded sample size with 519 tumor samples of HNSC and 44 normal tissue samples was revealed from the online database of GEPIA (Figure 1d). Using NCBI online database, differently expressed PRAME was also detected in diverse types of human tissues. Specifically, the expression of PRAME is low or absent in normal tissues except several anatomic sites, including testis, ovary, and adrenal gland (Figure 1e).

In this study, we also summarized the clinical information of LSCC patients (Tables 1 and 2). A chi-square test and a two independent samples’ *t*-test were conducted to further investigate the correlation of the expression level of PRAME with clinicopathological characteristics of LSCC patients. In 57 tumor tissues, the *P* values revealed the correlation between a high expression level of PRAME and the TNM staging (*P* < 0.05), as well as with lymphatic metastasis (*P* < 0.05) (Figure 1f and Table 2). However, no correlation was reported between the level of PRAME expression and age, cigarette smoking, or alcohol consumption (Figure 1f). Furthermore, it was explored whether the high expression of PRAME influenced the prognosis of laryngeal cancer patients. Kaplan–Meier analysis indicated that the progression-free survival (PFS) rate (Figure 1g) and the OS rate (Figure 1h) of patients in the PRAME high expression group were shorter than that in the PRAME low expression group. Altogether, the above data demonstrate that a high level of PRAME is correlated with poor prognosis in the LSCC patients, hinting that PRAME could be a carcinogenic factor for LSCC progression.

### 3.2 PRAME promotes the proliferation of LSCC cells *in vitro*

**Figure 2:**
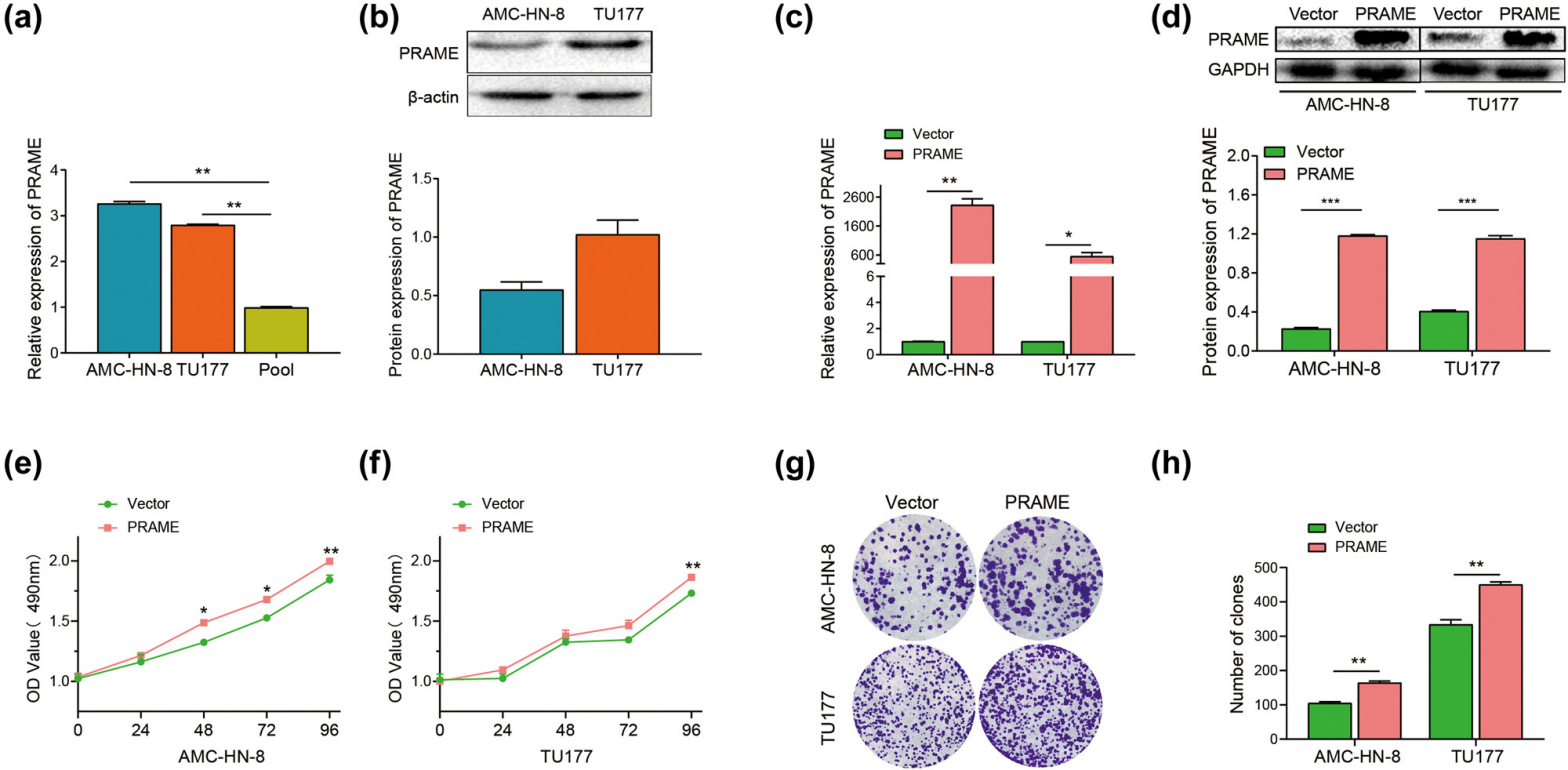
PRAME promotes the proliferation of LSCC cells *in vitro*. (a) PRAME mRNA levels were expressed in AMC-HN-8 and TU177 via qRT-PCR. (b) PRAME protein levels were expressed in AMC-HN-8 and TU177 via western blot assay. (c) The mRNA expression levels of PRAME significantly increased after the cells were transfected by means of qRT-PCR. (d) The protein expression levels of PRAME significantly increased after the cells were transfected by means of western blot assay. (e and f) PRAME obviously enhanced the proliferative capability via MTS assay. (g and h) PRAME obviously enhanced the colony formation capability (*P < 0.05, **P < 0.01, ***P < 0.001).

Given the potential oncogenic role of PRAME in the progression of LSCC, further study was designed to investigate the regulatory role of PRAME in LSCC cell proliferation *in vitro*. The expression levels of PRAME in LSCC were characterized in AMC-HN-8 and TU177. A higher mRNA expression levels of PRAME were observed in the cell lines of AMC-HN-8 and TU177 compared with the pool group (Figure 2a). The pool represents the mean value of relative expression of PRAME in the normal tissues, which, therefore, served as a control [33]. In addition, the protein expression levels of PRAME in both cells line of LSCC were identified by western blot assay (Figure 2b). To further elucidate the molecular function (MF) of PRAME in LSCC cells, AMC-HN-8 and TU177 were transfected respectively with overexpression of PRAME or the PcDNA3.1 empty vector. Both qRT-PCR (Figure 2c) and western blot assay (Figure 2d) indicated that the expression levels of PRAME were significantly increased after the cells were transfected for 48 h.

**Figure A1:**
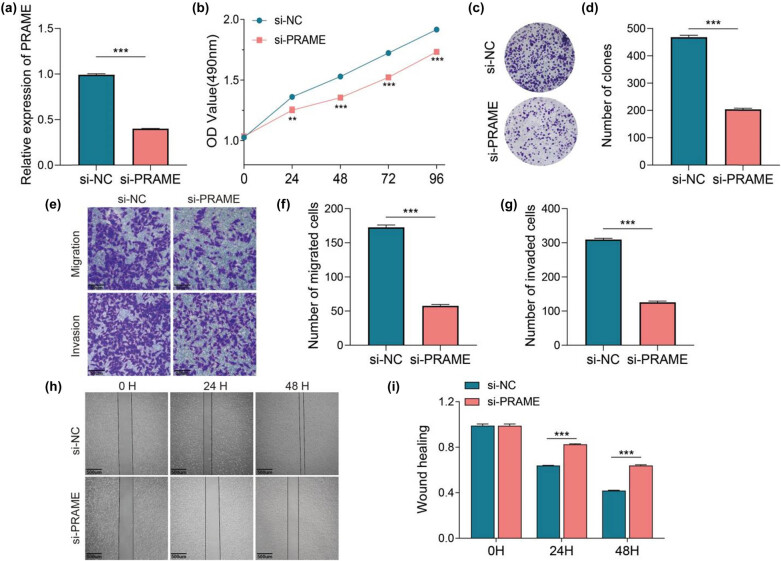
Downregulation of PRAME inhibits cell proliferation, migration and invasion *in vitro*. (a) The relative expression of PRAME significantly reduced after the cells were transfected with siRNA. (b) The cell viability was assessed via MTS assay after transfected with PRAME siRNA. (c and d) The colony formation capabilities were obviously decreased after PRAME was knocked down. (e–g) Decreased expression of PRAME attenuates the migratory and invasive capabilities by transwell assays. (h and i) The suppressed migration capability in cells treated with si-PRAME was demonstrated using wound healing assay . (*p < 0.05, **p < 0.01, ***p < 0.001).

After the transfection of PRAME in the AMC-HN-8 and TU177, the role of PRAME in the proliferation of LSCC was also investigated *in vitro*. MTS assay and colony formation assay were carried out to examine the function of PRAME on proliferation capabilities. MTS assay revealed that overexpression of PRAME obviously enhanced the proliferative capability of LSCC cell lines (Figure 2e and f). In addition, the number of AMC-HN-8 and TU177 colonies was significantly increased after overexpression of PRAME, indicating that PRAME enhances the colony formation capability in the LSCC cell lines of AMC-HN-8 and TU177 (Figure 2g and h). Since the expression of PRAME was high in the LSCC cell line of TU177, TU177 was respectively transfected by si-NC or PRAME siRNA to further elucidate the MF of PRAME. The qRT-PCR results of the transfection of siRNA of PRAME after 48 h indicated that the expression level of PRAME significantly decreased compared with the si-NC group (Figure A1a). After the transfection of PRAME siRNA, the role played by PRAME in the proliferation of TU177 was studied *in vitro*. MTS assay revealed that knock-down of PRAME obviously reduced the proliferative capability of LSCC cell lines (Figure A1b). In addition, the number of TU177 colonies showed an apparent decrease after the knock-down of PRAME (Figure A1c and d). The above findings reveal that PRAME can stimulate the proliferation of LSCC cells.

### 3.3 PRAME promotes the migration and invasion of LSCC cells via regulating the EMT.

**Figure 3:**
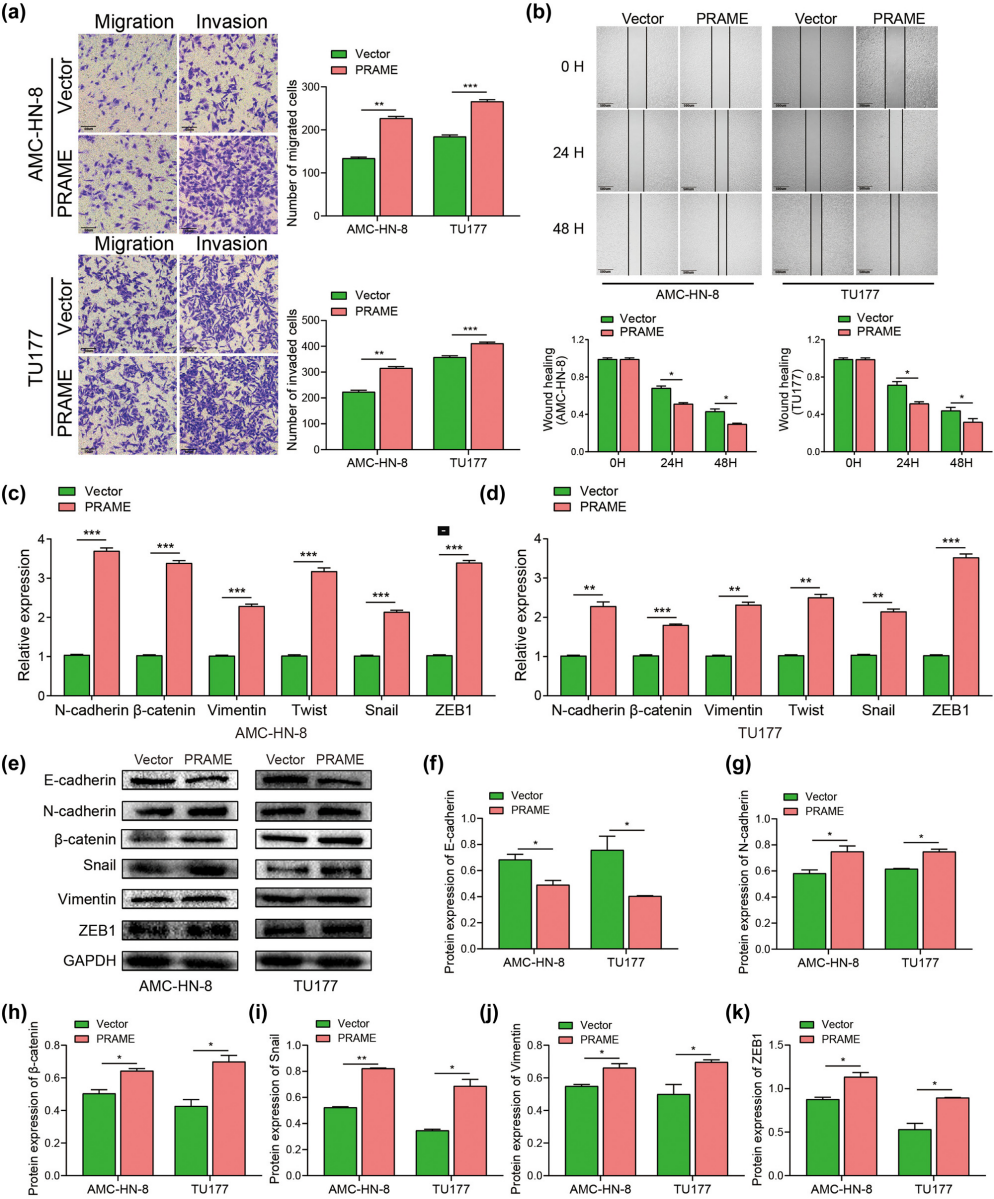
PRAME promotes the migration and invasion of LSCC cells via regulating the EMT. (a) PRAME obviously enhanced the migratory and invasive capabilities by transwell assays. (b) PRAME notably boosted the migration ability using wound healing assay. (c and d) PRAME promoted the mRNA levels of N-cadherin, β-catenin, Vimentin, Twist, Snail, and ZEB1 by means of qRT-PCR. (e–k) PRAME suppressed the protein expression of E-cadherin and promoted the protein expression of N-cadherin, β-catenin, Vimentin, Snail, and ZEB1 by means of western blot assay (*P < 0.05, **P < 0.01, ***P < 0.001).

Our clinicopathologic correlation analysis suggested that a high expression level of PRAME was correlated with the TNM staging and lymphatic metastasis. As a result, the following study was to explore the potential involvement of PRAME in the migration and invasion in LSCC cells via regulating the EMT. Transwell assays and wound healing assay were employed to determine the functions of PRAME on the migration and invasion capabilities of those cells. It can be seen that overexpression of PRAME obviously enhanced the invasive and migratory capabilities of LSCC cell lines in both of AMC-HN-8 and TU177, compared with the vector group, respectively (Figure 3a). Furthermore, the result of the wound healing assay indicated that PRAME enhanced the migration ability of AMC-HN-8 and TU177 at 24 and 48 h after the transfection with overexpressed PRAME, compared with the vector group (Figure 3b). In addition, it can be seen that the knock-down of PRAME significantly reduced the migratory and invasive capabilities of TU177 (Figure A1e–g). Furthermore, the wound healing assay showed that the knock-down of PRAME reduced the migration ability of TU177 at 24 and 48 h after the transfection with PRAME siRNA compared with the si-NC group (Figure A1h and i). Collectively, the aforementioned results indicate that PRAME can enhance the migratory and invasive capabilities of LSCC cells and may further contribute to understand the potential oncogenic role of PRAME in the advanced clinical stage and metastasis of LSCC patients.

Existing research reveals that the migration and invasion of cells are primarily dependent on the EMT process [34,35], in which PRAME-induced EMT of LSCC cells was investigated in the following experiment. The results suggested that the overexpression of PRAME significantly upregulated the mRNA expression levels of N-cadherin, Vimentin, β-catenin, Snail, Twist, and ZEB1 in the LSCC cell lines of AMC-HN-8 and TU177, compared with the vector group (Figure 3c and d). Moreover, AMC-HN-8 and TU177 transfected with overexpression of PRAME also showed a remarkable increase in the protein expression level of N-cadherin, β-catenin, Vimentin, Snail, and ZEB1, while overexpression of PRAME inhibited the protein expression level of E-cadherin in both cell lines compared with the vector group (Figure 3e–k). The above results reveal that EMT may mediate PRAME-induced migration and invasion of LSCC cells, thus facilitating the development of LSCC.

### 3.4 HDAC5 is underexpressed and negatively correlated with PRAME in LSCC

**Figure 4:**
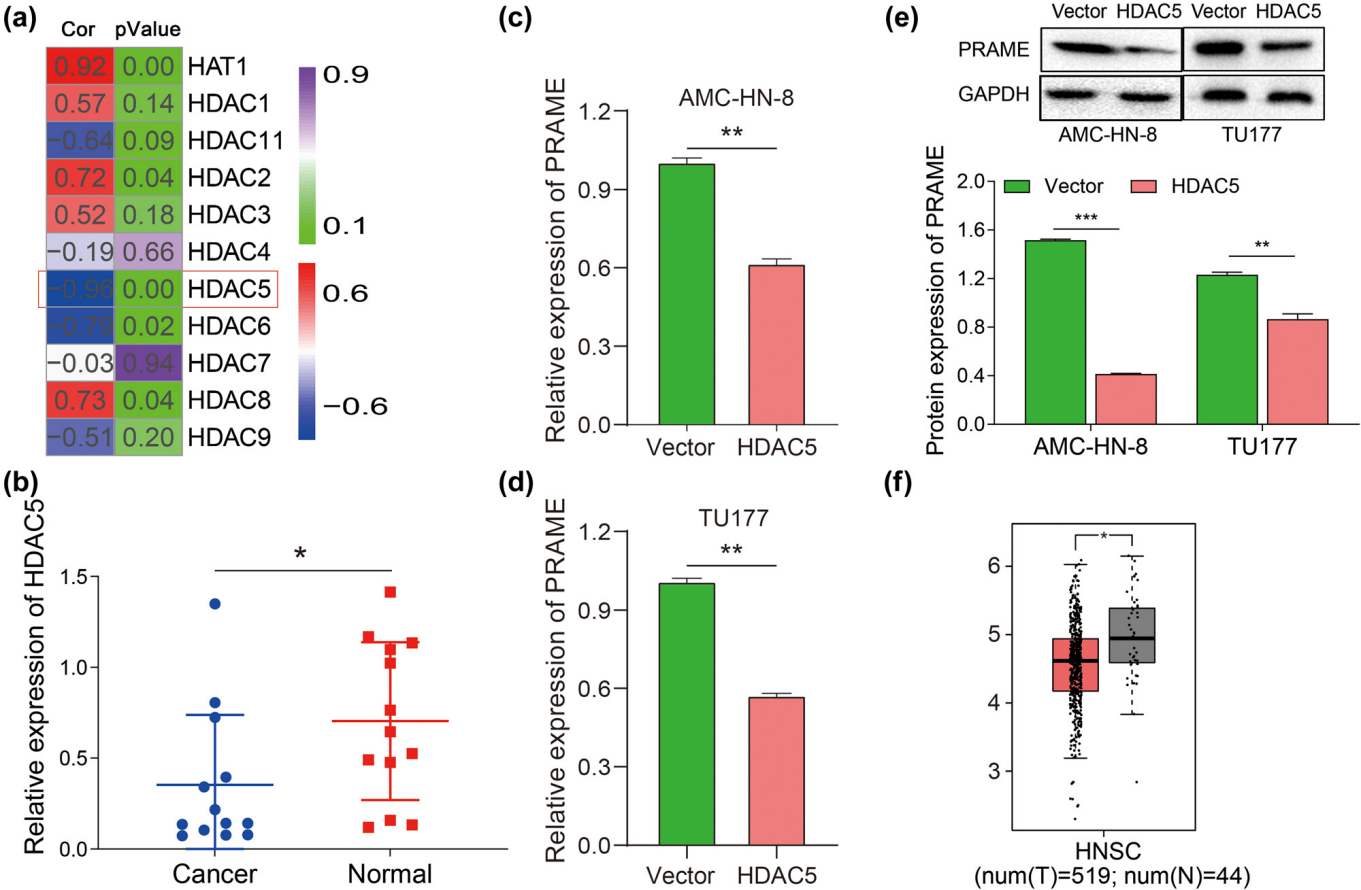
HDAC5 is underexpressed and negatively correlated with PRAME in LSCC. (a) HDAC5, as an important epigenetic regulator, was selected through the microarray analysis and was negatively correlated with PRAME. (b) HDAC5 expression was markedly downregulated, compared with corresponding normal tissues via qRT-PCR. (c) PRAME expression was significantly downregulated in AMC-HN-8 treated with overexpressed HDAC5 via qRT-PCR. (d) PRAME expression was significantly downregulated in TU177 treated with overexpressed HDAC5 via qRT-PCR. (e) The protein expression of PRAME was significantly reduced in AMC-HN-8 and TU177 cells treated with overexpressed HDAC5 by means of western blot assay. (f) The expression of HDAC5 were significantly lower in HNSC, compared with paired non-tumor tissues, cited from GEPIA (*P < 0.05, **P < 0.01, ***P < 0.001).

HDACs are the family of important epigenetic regulators, contributing to the regulation of gene expression through histone acetylation. Increasing evidence reveals that the abnormal expression of HDACs can play a role in various tumors, which is the mechanism of carcinogenic effects to cancer development and progression [36]. In this study, microarray analysis was conducted to identify the HDAC subtypes also expressed in LSCC tissues. Interestingly, the correlation analysis between PRAME expression and molecular subtypes of HDACs suggested that HDAC5 expression was negatively correlated with PRAME expression in the study (Cor = −0.96, *P* = 0.00) (Figure 4a). To be specific, gene expression analysis indicated significant downregulation of HDAC5 expression in LSCC tissues compared with corresponding normal tissues (Figure 4b). Moreover, qRT-PCR results showed that the relative level of PRAME mRNA expression in both LSCC cell lines of AMC-HN-8 and TU177 were significantly downregulated in HDAC5 overexpression group, compared with the vector group (Figure 4c and d). Furthermore, the same trend had also been observed that protein level of PRAME was downregulated in AMC-HN-8 and TU177 cells by manipulating HDAC5 genes (Figure 4e). In addition, an expanded sample size including 519 tumor samples of HNSC and 44 normal tissue samples was adopted to examine the expression of HDAC5 in the GEPIA online database. The expression of HDAC5 was lower in HNSC than in normal tissue (Figure 4f). In brief, the above results suggest that HDAC5 may play a role in regulating PRAME expression, thus facilitating the progression of LSCC.

### 3.5 PRAME promotes LSCC progression via the PI3K/AKT/mTOR signaling pathway

**Figure 5:**
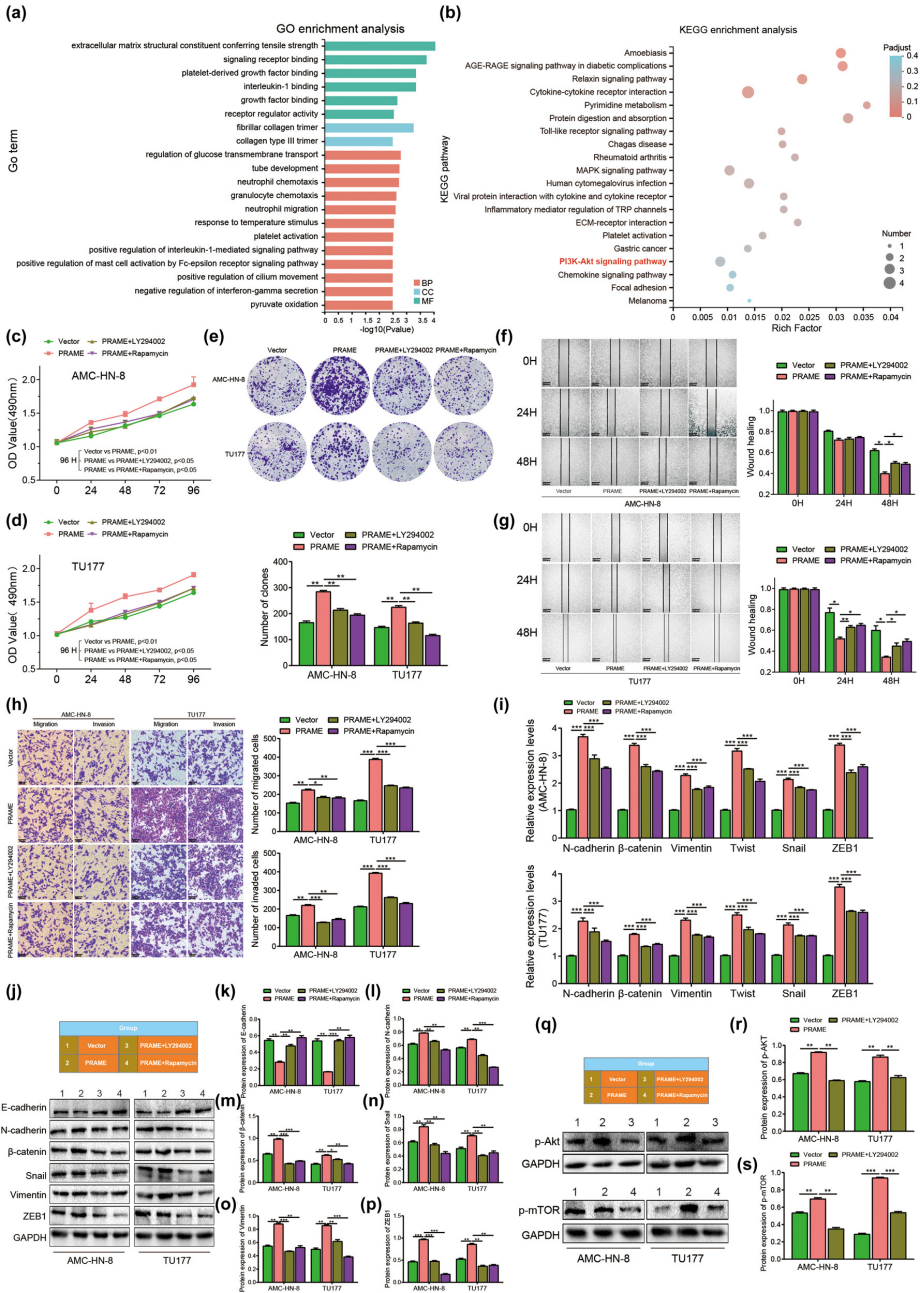
PRAME promotes the progression of LSCC via the PI3K/AKT/mTOR signaling pathway. (a) The relevant biological function of PRAME through the GO enrichment analyses. (b) The expression of PRAME showed strong correlation with PI3K/AKT/mTOR signaling pathway through the KEGG enrichment analyses. (c–e) The inhibitor (LY294002 or Rapamycin) partially abrogated PRAME overexpression-induced cell proliferation *in vitro*. (f–h) The inhibitor (LY294002 or Rapamycin) partially abrogated PRAME overexpression-induced cell migration and invasion *in vitro*. (i) The inhibitor (LY294002 or Rapamycin) abrogated PRAME overexpression-induced mRNA expression levels of EMT markers. (j–p) The inhibitor (LY294002 or Rapamycin) abrogated PRAME overexpression-induced protein expression levels of EMT markers. (q–s) LY294002 or Rapamycin reversed the protein expressions of p-AKT and p-mTOR mediated by PRAME (*P < 0.05, **P < 0.01, ***P < 0.001).

To explore the underlying mechanism of PRAME in the progression of LSCC, enrichment of functions of Gene Ontology (GO) and KEGG pathway analyses were conducted to predict downstream signaling pathways. This study was conducted based on GO term analysis with respect to biological process (BP) and cellular component (CC), as well as MF. The functional enrichment results revealed that 12 BP terms, 6 MF terms, and 2 CC terms were correlated with the overexpression of PRAME (Figure 5a). Interestingly, it was found that the expression of PRAME was significantly correlated with mitogen-activated protein kinase signaling pathways and its downstream of PI3K/AKT/mTOR signaling pathway (Figure 5b). To verify whether the PI3K–AKT signaling pathway is a result of PRAME activation, whether pharmacological inhibition of this pathway can sensitize LSCC cell lines to overexpressed PRAME induction was tested. Indeed, the experimental results showed that treatment with PI3K inhibitor LY294002 or mTOR inhibitor Rapamycin could partially abrogate PRAME overexpression-induced cells’ proliferation, migration, and invasion *in vitro* (Figure 5c–h).

Furthermore, PRAME overexpression-induced mRNA or protein expression levels of EMT markers of E-cadherin, β-catenin, N-cadherin, Snail, Vimentin, and ZEB1 were also partially abrogated in this pathway (Figure 5i–p). In the subsequent studies, western blot assay was used to further identify the potential signaling pathway correlated with the upregulation of PRAME. The result revealed increased protein expression levels of p-AKT and p-mTOR with the high expression level of PRAME, while those protein levels induced by PRAME were also decreased after treatment with the inhibitor LY294002 or Rapamycin (Figure 5q–s). Therefore, the above results suggest that the proliferation, migration, and invasion of LSCC cells that are induced by PRAME may be at least partially dependent on the activation of PI3K/AKT/mTOR signaling pathways.

### 3.6 PRAME promotes the proliferation of LSCC *in vivo*

**Figure 6:**
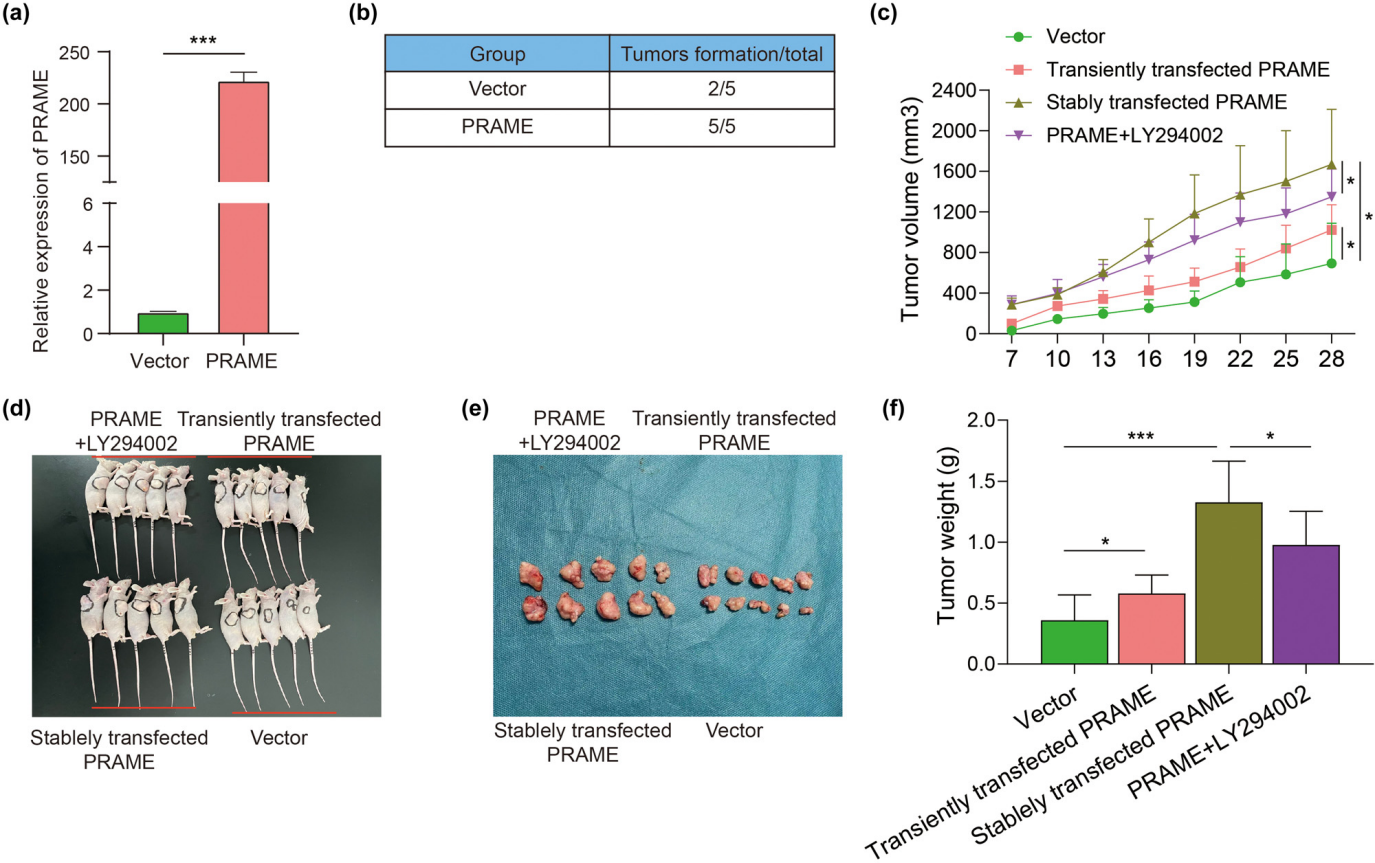
PRAME promotes the proliferation of LSCC *in vivo*. (a) The expression levels of overexpressed PRAME were significantly higher in AMC-HN-8 by means of qRT-PCR. (b) All of five athymic Nu/Nu mice were inoculated by the AMC-HN-8 cells transiently transfected with overexpressed PRAME grew tumors, while only two mice in the empty vector group formed any palpable tumors after one week. (c) Tumor growth rate was evidently increased in the stably transfected PRAME group relative to the empty vector group, and tumors’ growth rate with LY294002 was decreased compared with the stably transfected PRAME group. (d) The figure of tumor dimensions in nude mice at the end of the experiment. (e and f) The size and weight of the tumors in the stably transfected PRAME group were significantly increased relative to the empty vector group, while the size and weight of the tumors with LY294002 were decreased (*P < 0.05, **P < 0.01, ***P < 0.001).

To further verify the tumorigenic ability of PRAME *in vivo*, the mice were subcutaneously inoculated with AMC-HN-8 cells stably transfected with overexpressed PRAME. Importantly, there was a significant increase in the relative expression levels of PRAME in AMC-HN-8 cells after the transfection with overexpressed PRAME (Figure 6a), suggesting that high viability of AMC-HN-8 cells contributed to the mouse xenograft models for LSCC. As a control, the AMC-HN-8 cell line with stable pcDNA3.1 was constructed. At 1 week after tumor cell transplantation, tumors formed in all of five athymic Nu/Nu mice by PRAME-overexpressed AMC-HN-8 cells, while only two mice developed tumors in the empty vector group (Figure 6b).

Therefore, the effect of overexpressed PRAME on the growth of primary tumor xenografts was investigated in nude mice. In addition, tumor volume was documented every week. As depicted in Figure 6c, the tumor-bearing mice administered by stable transfection with overexpressed PRAME showed a rapid tumor volume growth on day 28. In contrast, other three groups showed the significantly delayed tumor growth at 4 weeks after the transplantation (Figure 6c). To further confirm that PRAME regulates the proliferation of LSCC through the PI3K/AKT/mTOR signaling pathways *in vivo*, tumors were treated with LY294002 at 1 week after transplantation, 15 mg/kg for each mouse, once every 3 days. An important finding was that PI3K inhibitor LY294002 administered 1 week after stable transfection with overexpressed PRAME demonstrated an effective tumor inhibition (Figure 6c). The results indicated that the tumors’ growth rate was decreased compared with the only stable transfection with the overexpressed PRAME group (Figure 6c).

After 4 weeks, the nude mice were sacrificed and then tumor xenografts were harvested. At the end of the experiment, there was a clear figure of tumor dimensions in nude mice among the four groups (Figure 6d). In addition, the photograph of the excised tumors and their weight were significantly different among the four groups at the end of the experiment (Figure 6e and f). The tumor weight of the stable transfection with the overexpressed PRAME group was significantly higher than that in other three groups. All of the results reveal that PRAME facilitates the proliferation of LSCC *in vivo*, which may be due to the activation of PI3K/AKT/mTOR signaling pathways.

## 4 Discussion

In this study, we found that PRAME expression was significantly correlated with the clinical significance in the LSCC patients. PRAME expression was markedly upregulated in LSCC tissues compared with corresponding normal tissues. GEPIA and NCBI online database were also adopted to confirm the presence of high PRAME expression in the LSCC patients. Notably, this study found that high level of PRAME expression was correlated with the OS rate and the PFS rate of LSCC patients, and PRAME may serve as a poor survival biomarker candidate. The *in vitro* result suggested that PRAME overexpression can stimulate LSCC-related AMC-HN-8 and TU177 cell proliferation, migration, invasion, and EMT. In addition, the result showed that the knock-down of PRAME significantly reduced the proliferative, migratory, and invasive capabilities of TU177. The *in vitro* result also revealed that HDAC5, as a potential upstream regulator, may mediate PRAME expression, thus facilitating the progression of LSCC. Furthermore, our *in vitro* and *in vivo* data revealed that LSCC cell proliferation, migration, and invasion induced by PRAME may be at least partially dependent on the activation of PI3K/AKT/mTOR signaling pathways. The above findings provide insights into the function of PRAME in LSCC development and progression, providing the new evidence for PRAME as an oncogene that may be a valuable target for the diagnosis and treatment of LSCC.

Although the epigenetics of LSCC and its underlying molecular mechanisms have been extensively investigated over the past few years, there are still unsurmountable challenges in high incidence, high invasion, high metastasis, local recurrence, and poor prognoses [37]. Surgery resection is still the first-line therapeutic strategy since the underlying molecular mechanism of LSCC remains unclear. Existing studies had implicated that the function of PRAME has been mainly correlated with melanoma and several hematological cancers [38,39]. However, increasing evidence suggests that PRAME is linked to a cancer-promoting gene in various types of cancers. Especially, high PRAME expression plays an important role in head and neck squamous cell carcinoma, which is expected to be a novel therapeutic target and a biomarker of poor outcome [40,41]. More specifically, our study further confirmed such findings that PRAME was highly expressed in the LSCC patients, with potentially poor outcome, which may also contribute to the development of LSCC. In addition, it is reported that PRAME is closely correlated with disease recurrence patterns and survival rates in esophageal squamous cell carcinoma (ESCC) [15]. Nevertheless, the diagnostic and prognostic value of PRAME in LSCC should be further explored with large-scale studies in the future.

To examine the biological functions of PRAME in contribution to the development as well as progression of LSCC, this *in vitro* study found that PRAME in LSCC was also identified in the LSCC cell lines AMC-HN-8 and TU177. In our *in vitro* experiments, we also found that PRAME promoted AMC-HN-8 and TU177 cell proliferation, migration, and invasion by the regulation of EMT. Furthermore, the results of the *in vivo* experiments on tumor xenografts in nude mice supported *in vitro* experiments, in which tumor-bearing mice administered by transfection with overexpressed PRAME displayed a rapid tumor volume growth on day 28. It is well known that EMT has been considered to be required for cell proliferation, migration, and invasion in various human cancers [42,43,44]. During the EMT process, tumor cells lose the epithelial properties and gain mesenchymal phenotypes, and there are an upregulation of the expression of mesenchymal markers and a downregulation of the expression of epithelial markers [45,46]. This study reveals that overexpression PRAME plays a role in the regulation of EMT and changes the expression of the above EMT-related markers. The above results showed that overexpression PRAME could result in a downregulation of the expression of epithelial-related markers, including E-cadherin, and an increase in the expression of mesenchymal markers including N-cadherin, vimentin, snail, twist, ZEB1, and β-catenin [47]. In terms of mechanism, cooperation of different transcription factors such as snail, twist, and ZEB1 induces the orchestration of the cadherin switches that contributes to alteration of cell adhesion properties during EMT, promoting mesenchymal morphology [48]. Taken together, we have preliminarily found that PRAME upregulation drives the EMT process and hence promotes LSCC cell proliferation, migration, and invasion via modulating EMT-related gene expression.

Studies have found that EMT process is regulated by a wide variety of signaling pathways. The PI3K/AKT/mTOR signaling pathways have been known to be implicated in tumorigenic roles in various cancers via the EMT process [49]. Existing research has suggested that inhibiting PI3K/AKT/mTOR and EMT markers can suppress the invasion and migration of cancer cells in lung cancer, liver cancer, as well as breast cancer [50,51,52]. With respect to the complexity and diversity, the critical regulatory mechanism of the EMT process driven by PRAME in LSCC was also validated between *in vitro* and *in vivo* study. In our *in vitro* study, PI3K inhibitor LY294002 or mTOR inhibitor Rapamycin inhibited the PRAME overexpression-induced cells’ proliferation, migration, and invasion in the LSCC cell lines of AMC-HN-8 and TU177. Moreover, EMT process enhanced by the PRAME overexpression was seriously weakened after treatment with the two inhibitors targeting on the PI3K/AKT/mTOR pathway, as indicated by the variation of EMT-related markers, including E-cadherin, N-cadherin, β-catenin, Snail, Vimentin, and ZEB1, consistent with other similar studies. LY294002, an inhibitor of PI3K, can suppress the EMT in hepatocellular carcinoma (HCC) cell lines of Huh-BAT and HepG2 [49]. TGFβ-induced EMT and cell migration in breast tumor cell of 4T1 and EMT6 were also inhibited by LY294002 or Akt mutants through PI3K-Akt signaling [53]. In addition, other studies confirmed that EMT promoted tumorigenesis through the PI3K/Akt/mTOR signaling in colorectal cancer and brain metastasis of breast cancer that were reversed by treatment with mTOR inhibitor Rapamycin [54]. It is noteworthy that PRAME has previously been confirmed to play a role in promoting EMT in ESCC and breast cancer, and it is correlated with malignant behavior of the proliferation, migration, and invasion [15,17,55]. Thus, our results confirmed the previous findings, and this study reveals that PRAME facilitates LSCC cell proliferation and highly malignant phenotype, through activation of PI3K/AKT/mTOR signaling pathways. Notably, our *in vivo* study further strengthens the view that PI3K/AKT/mTOR signaling plays a role in LSCC in the development and progression of LSCC.

Histone acetylation is a typical type of epigenic modification, and the level and state are regulated by both HATs and HDACs [56,57]. HATs and HDACs work together to maintain the dynamic balance of histone acetylation, while disruption of the balance could affect cell proliferation and apoptosis, consequently leading to tumor occurrence [58,59]. HDAC5, a class of HDAC, was reported to have diverse expressions and functions in terms of various types of tumors. For instance, overexpressed HDAC5 is reported in breast cancer [60,61], lung cancer [62], HCC [63], pancreatic neuroendocrine cancer [64], and colorectal cancer (CRC) [65]. In contrast, HDAC5 is decreased in gastric cancer [66]. This study found that HDAC5 expression was downregulated in LSCC. In addition, HDAC5 in GEPIA online database adopted showed a low level in HNSC. HDAC5 has been found to play a role in multiple biological functions (e.g., cell proliferation, apoptosis, metastasis, and invasion), in response to immune regulation, cell differentiation and stemness, and drug resistance, suggesting that it may be a potentially useful biomarker for diagnosis and prognosis of cancer [67]. Moreover, this study confirms that there is a negative correlation of the expression of PRAME with HDAC5 and PRAME expression is downregulated under HDAC5 overexpression. As revealed by the *in vitro* study in human acute myeloid leukemia cells, a selective HDAC inhibitor chidamide can upregulate PRAME mRNA expression [68]. It was also reported that the suppressive effect of HDAC inhibitors (HDACi)-induced cell proliferation could be blocked by the ectopic expression of PRAME in colony formation assays [69]. Thus, it is reasonable to speculate that HDAC5 may mediate the expression of PRAME, contributing to the progression of LSCC. In contrast, it has also been shown that HDAC-independent mechanism (e.g., retinoic acid receptor signaling) regulates PRAME expression, thus leading to the development of cancer [70]. Further investigation in specific targeting of PRAME expression by HDAC5 is required. However, the fact that our study did not delve into a further in-depth regulation mechanism is one of the limitations in this study. Of course, there are still other some shortcomings in this study. For instance, we should enlarge the sample size to support the presence of high PRAME expression in LSCC patients. At the same time, it is essential to increase multi-centre studies, evaluating the PRAME expression. We did not use the Akt inhibitors like GSK 690693 to confirm the PRAME overexpression-induced malignant behavior in the LSCC cell lines and primary tumor xenografts, contributing to enhance our hypothesis that PI3K/AKT/mTOR is involved in LSCC development. Moreover, we did not explore additional signaling pathways like IL-17 signaling [71] that could contribute to the progression of LSCC from an immunological point of view.

**Figure 7:**
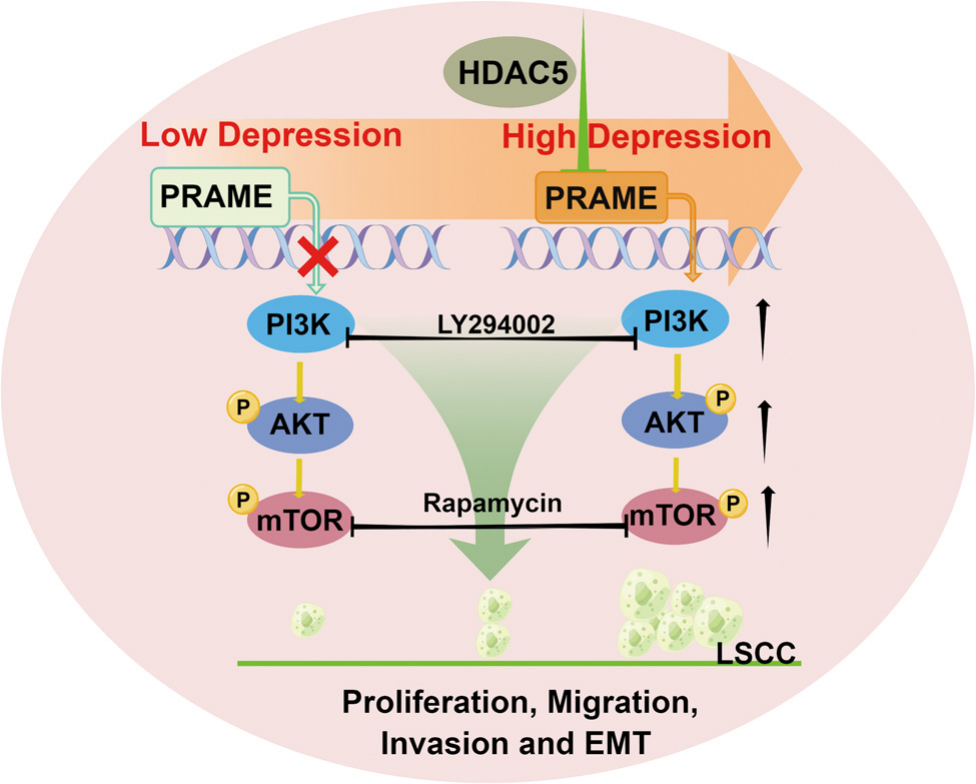
Schematic diagram summarizing the function of PRAME and PRAME-induced PI3K/AKT/mTOR signaling pathway.

In the present study, we found that PRAME was overexpressed in the LSCC patients, and which was also significantly correlated with the TNM staging and lymphatic metastasis. *In vitro* studies confirmed that PRAME facilitated the proliferation, invasion, migration, and EMT of human LSCC cell lines. *In vivo* also substantiated that PRAME promoted tumor growth. In addition, HDAC5 was identified as an upstream regulator that can affect the expression of PRAME and contribute to LSCC progression. Moreover, both *in vitro* and *in vivo* studies further confirmed that PRAME can facilitate the proliferation, migration, invasion, and EMT of LSCC cells and promote tumor growth, at least partially by activating PI3K/AKT/mTOR pathways (Figure 7). In brief, it is suggested that PRAME may be a valuable oncogene target, contributing to the clinicopathologic features in the LSCC patients.

## Abbreviations

LSCC: laryngeal squamous cell carcinoma;
ESCC: esophageal Squamous cell carcinoma
PRAME: preferentially expressed antigen in melanoma
GO: Gene Ontology
KEGG: Kyoto Encyclopedia of Genes and Genomes
EMT: epithelial–mesenchymal transition
HATs: histone acetyltransferases
HDACs: histone deacetylases
